# Biological efficacy of zinc oxide nanoparticles against diabetes: a preliminary study conducted in mice

**DOI:** 10.1042/BSR20193972

**Published:** 2020-04-07

**Authors:** Shafayet Ahmed Siddiqui, Md. Mamun Or Rashid, Md. Giash Uddin, Fataha Nur Robel, Mohammad Salim Hossain, Md. Azizul Haque, Md. Jakaria

**Affiliations:** 1 Department of Pharmacy, Noakhali Science and Technology University, Sonapur, Noakhali 3814, Bangladesh; 2 Department of Pharmacy, University of Chittagong, Chittagong 4331, Bangladesh; 3 Department of Applied Chemistry and Chemical Engineering, Noakhali Science and Technology University, Sonapur, Noakhali 3814, Bangladesh; 4 Melbourne Dementia Research Centre, The Florey Institute of Neuroscience and Mental Health, The University of Melbourne, Parkville, VIC 3010, Australia

**Keywords:** antidiabetic, insulin, oral glucose tolerance test, streptozotocin, ZnONPs

## Abstract

The antidiabetic, hypoglycemic and oral glucose tolerance test (OGTT) activities of zinc oxide nanoparticles (ZnONPs) were assessed in mice. ZnONPs were prepared by reacting Zn(NO_3_)_2_.6H_2_O and NaOH solution at 70°C with continuous stirring and then characterized by transmission electron microscopy (TEM) and scanning electron microscopy (SEM) techniques. Diabetes was induced by the intraperitoneal injection of streptozotocin (STZ) in mice, and then the blood glucose levels were determined by the glucose oxidase method. The experimental results revealed that ZnONPs suggestively (*p*<0.001) declined the blood glucose levels (39.79%), while these reductions were 38.78% for the cotreatment of ZnONPs and insulin, and 48.60% for insulin, respectively. In the hypoglycemic study, ZnONPs (8 and 14 mg/kg b.w) reduced approximately 25.13 and 29.15% of blood glucose levels, respectively. A similar reduction was found in the OGTT test, which is also a dose- and time-dependent manner. Overall, ZnONPs possess a potential antidiabetic activity, which could be validated by further mechanistic studies.

## Introduction

The epidemic metabolic disorder, diabetes mellitus (DM) is characterized by high blood glucose where the patient cannot produce adequate insulin, or body cells do not respond to glucose produced by β-cells in the pancreas. Many people have diabetes all over the world [1–3], and the number is increasing at an alarming rate. According to the World Health Organization, diabetes causes 1.5 million deaths each year worldwide, being one of the most recurrent cause of mortality in developed countries [2]. The high blood sugar of diabetic patients leads to the typical symptoms of polyuria, polydipsia and polyphagia [4]. The development of several medications with multiple modes of actions having glucose-lowering activity is needed to manage diabetes. Many researchers have proved that the role of trace metals with glucose metabolism and their relationship with diabetes. Zinc [5], magnesium [6] and chromium [7] have reported to play a role in blood sugar maintenance and used in diabetes therapy. Over 300 enzymes are activated by zinc in the body, and it plays a crucial role in different metabolic pathways, including glucose metabolism [8]. Zinc is also known to keep the structure of insulin [9] and plays a vital role in insulin biosynthesis, storage and secretion [10]. It has been proved by researches that several zinc transporters in pancreatic β-cells like zinc transporter-8 have a potent role in insulin secretion [11,12]. By following numerous mechanisms, including increased phosphorylation of insulin receptor, enhanced phosphoinositide 3-kinases activity and inhibited glycogen synthase kinase-3, zinc also could improve insulin signaling [13]. Therefore, there is a very complex inter-relationship between zinc, diabetes and diabetic complications and related diabetic symptoms.

Simpler and painless routes for insulin administration are still in demand to manage diabetes as conventional drug delivery systems still have numerous drawbacks, including improper and/or ineffective dosage, low potency, limited specificity for the target, which may result in adverse side effects in other organs/tissues. The use of natural products could be the potential to encounter the limitations of DM treatment [2,14,15]. To overcome the pharmacokinetic limitations of drugs, the novel drug delivery system has considered as an attractive option to deliver the drugs for treating several diseases and disorders [2,16–18]. The loading of insulin, and other sugar-lowering drugs and nutraceuticals, into nanoparticles, has been proposed as a more convenient, non-invasive and safer approach through alternative administration routes [2].

Zinc oxide nanoparticles (ZnONPs), a novel agent to deliver zinc, have great implications in many disease therapies including DM [19]. The development of zinc-based agent would be promising in the treatment of diabetes and its associated complications as zinc supplement have shown ameliorating effect in preclinical studies [19,20]. Alkaladi et al. (2014) [21] studied the antidiabetic role of ZnONPs through induction of insulin, insulin receptors and glucose metabolizing enzymes gene expression. Umrani and Paknikar [22] proved the role of ZnONPs for controlling blood glucose in diabetic rats in the same line. In both studies, they monitored the effect of ZnONPs on diabetic rats only; therefore, in the present study, we tested ZnONPs to validate its effect against diabetes; therefore, we used the mouse models to assess its hypoglycemic and glucose tolerance effects as its effect is already studied in the rat models.

## Materials and methods

### Materials

Streptozotocin (STZ; Sigma Chemical Co. Ltd., U.K.), NaOH (Loba Chemie, India), ZnO (Loba Chemie, India). ACCU-CHEK active blood glucose meter (Roche Diagnostics Ltd, U.K., serial #GU 02591300). All other chemicals were of analytical grade and obtained from the Laboratory of the Department of Pharmacy, Noakhali Science and Technology University, Bangladesh.

#### Experimental animals

A total number of 60 mature Swiss albino mice (weight: 22–25 g) were used in the present study. Mice were obtained from the animal house of Jahangirnagar University (JU), Bangladesh. Mice were housed in separate metal cages while maintaining constant environment and nutritional conditions. Fresh and clean drinking water was supplied *ad libitum* through the specific nipple. The cages of the mice were cleaned regularly, and water and food were supplied twice daily. Acclimatization was done for 7 days before conducting the experiments. All animal experiments were conducted at the Department of Pharmacy, Noakhali Science and Technology University, Bangladesh.

### Synthesis of ZnONPs

ZnONPs were prepared by using the method of Gusatti et al. (2011) [23] with slight modification. First, 0.5 M ZnNO_3_ hexahydrate solution and 1 M NaOH solution were prepared, and the necessary solution was heated up to 70°C and stirred continuously with a magnetic stirrer. Then, ZnNO_3_ hexahydrate solution was added dropwise into the primary solution slowly with continuous stirring. A white solution of ZnO was observed; in fact, the nanoparticles were dispersed into this white solution. After finishing the addition of ZnNO_3_ hexahydrate, the mixture was stirred for 2 h more which was followed by cooling. Prepared ZnONPs were filtered and washed by deionized water. Finally, the ZnONPs containing suspension were dried at 65°C for obtaining the white solid residue. These particles were characterized before starting our experiments.

#### Characterization studies

Transmission electron microscopy (TEM) (JEM 1400 plus, JEOL Ltd., Japan) and scanning electron microscopy (SEM) (JCM 5700, JEOL Ltd., Japan) was employed to determine the size, shape and morphology of ZnONPs. The study was conducted at the Departmental Laboratory of Applied Chemistry and Biochemistry, Kumamoto University, Japan.

### Induction of experimental diabetes

Multiple intraperitoneal (i.p.) injection (45 mg/kg body wt.) of STZ was used to induce diabetes in experimental mice. STZ was dissolved in freshly prepared 0.01 M sodium citrate buffer (pH = 4.5) and administered on mice for 5 consecutive days. After STZ injection, the animals could drink glucose solution (5% w/v) overnight to avoid hypoglycemia which might be induced by STZ. Control mice received an equivalent amount of vehicle (citrate buffer) only. When the dosing was completed, STZ-treated mice were kept in normal conditions for 5 days. In fact, after this duration, mice had developed diabetes (blood glucose levels ≥ 11.1 mmol/l) at fasting conditions. The STZ-treated mice were fastened for 12 h, and blood samples were collected from the tail vein for determining the blood glucose levels. Mice in the diabetic group with fasting blood glucose levels higher than 11.1 mmol/l were considered as diabetic, and hence they were selected for further studies.

### Animal grouping

For the antidiabetic activity test, the diabetic mice were randomly subdivided into four groups containing five mice in each group. We have followed the experimental procedure of Alkaladi et al. (2014) for this test [21]. Mice were grouped as G-I group for control, where mice were only treated with distilled water. G-II (ZnONPs treated), G-III (ZnONPs and insulin-treated) and G-IV stand for the standard treated group where diabetic mice were injected with insulin 0.4 U/50 mg subcutaneously (s.c.). Each group received their respective doses for consecutive 14 days.

In the hypoglycemic study, 20 mice were taken and grouped into four groups and test was done according to the method of Farag et al. (2014) [24]. Grouping of mice were done as: G-I (control: distilled water, 1 ml/mice, oral), G-II (ZnONPs: 8 mg/kg b.w., oral), G-III (ZnONPs: 14 mg/kg b.w., oral) and G-IV (standard: glibenclamide, 10 mg/kg b.w., oral).

In terms of oral glucose tolerance test (OGTT), we used the method described by Andrikopoulos et al. (2008) [25]. Mice were given 2 g/kg glucose levels orally after 6 h of fasting, and glucose was given orally a few minutes before ZnONPs dosing. Blood glucose was measured at 0, 15, 30, 60, 90 min. Mice were grouped into G-I (control: distilled water, 1 ml/mice, oral), G-II (ZnONPs: 8 mg/kg b.w., oral), G-III (ZnONPs: 14 mg/kg b.w., oral) and G-IV (standard: glibenclamide, 10 mg/kg b.w., oral), where each group contained five mice. Mice were not subjected to killing as tissues were not investigated in the present study.

### Biochemical determination

By following the glucose oxidase method using the ‘ACCU-CHEK Active’ kit, the blood glucose levels were determined. The blood glucose levels were measured in all experimental animals before the beginning of the experimental procedures. Fasting blood glucose levels were routinely measured until diabetes was confirmed (mice with fasting blood glucose ≥ 11.1 mmol/l was considered as diabetic).

#### Sampling protocol

Blood samples (2–3 µl blood) were collected from the tail vein of all experimental mice. The blood glucose levels were monitored by ACCU-CHEK active blood glucose meter.

### Statistical analysis

Data were analyzed using one-way ANOVA tests (SPSS software, version-20) followed by Dunnett’s *t* tests. *p≤0.05 was considered statistically significant, **p≤0.01 moderately significant and ***p≤0.001 highly statistically significant. Values were expressed as mean ± standard error of the mean (S.E.M.) Origin Pro (ver. 8.5, Origin Lab. Corp., U.S.A.) was used for preparing graphical representations.

## Results

### Characterization of ZnONPs using SEM and TEM imaging

**Figure 1:**
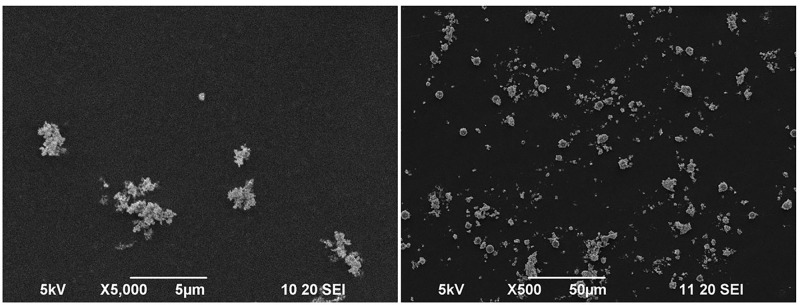
SEM image of ZnONPs. Synthesized nanoparticles were <100 nm in most of the cases; however, few particles are more abundant This may happen due to the aggregation of smaller particles after synthesis.

**Figure 2:**
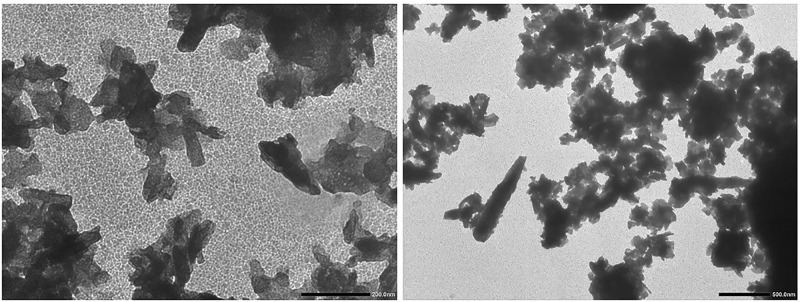
TEM image of ZnONPs Most of the nanoparticles were non-spherical; however, some are roughly circular. Most of the synthesized nanoparticles were less than 100 nm in size.

High-resolution SEM and TEM imaging were performed for getting the information about structural morphology, size and shape of the synthesized ZnONPs. It was found that most of the ZnONPs were non-spherical; however, some of them were roughly circular (Figures 1 and 2). The size of synthesized nanoparticles was less than 100 nm in most of the cases. However, few particles were more significant than this. The larger ZnONPs found in SEM measurement may be due to the accretion of the smaller ones.

### Assessment of antidiabetic activity of ZnONPs

The therapeutic effect of ZnONPs on STZ-induced diabetic mice, as well as their compared effect on insulin treatment, were assessed. The results showed a significant reduction in blood glucose levels in the mice of G-II (ZnONPs treated) and G-III (ZnONPs and insulin-treated) when compared with diabetic non-treated mice, G-I (control) Table 1.

**Table 1:** Antidiabetic effect of ZnONPs on STZ-induced diabetic mice

Groups	Blood glucose levels (mmol/l)			
	Dose	Fasting glucose levels	Glucose levels after 2 h	% of inhibition
G-I (Control: Distilled water)	1 ml/mice (oral)	18.68 ± 0.34	18.54 ± 0.16	-
G-II (ZnONPs)	14 mg/kg b.w. (oral)	18.32 ± 0.22	11.04 ± 0.22*	39.74
G-III (ZnONPs + Insulin)	7 mg/kg b.w. (oral) + 0.2 U/50 g (s.c.)	18.62 ± 0.27	11.40 ± 0.21*	38.78
G-IV (Standard: Insulin)	0.4 U/50 g (s.c.)	18.60 ± 0.19	9.56 ± 0.32*	48.60

Mean ± S.E.M. (*n*=5). Data were analyzed using one-way ANOVA followed by Dunnett’s *t* test and compared with control.

* *P*<0.005 was considered significant.

#### Assessment of hypoglycemic activity of ZnONPs

It was found that a sharp and significant decrease in glucose levels were attained when mice were treated with ZnONPs mild dose (G-II: 25.13% inhibition) and ZnONPs moderate dose (G-III: 29.15% inhibition). Standard drug (glibenclamide) can reduce 49.25% reduction in blood glucose levels (G-IV) (Table 2).

**Table 2:** Hypoglycemic activity of ZnONPs

Groups	Blood glucose levels (mmol/l)			
	Dose	Fasting glucose levels at 0 h	Glucose levels after 2 h	% of inhibition
G-I (Control: Distilled water)	1 ml/ mice (oral)	4.10 ± 0.12	3.98 ± 0.06	-
G-II (ZnONPs, mild dose)	8 mg/kg b.w. (oral)	3.84 ± 0.19	2.98 ± 0.15*	25.13
G-III (ZnONPs, moderate dose)	14 mg/kg b.w. (oral)	4.12 ± 0.13	2.82 ± 0.12^†^	29.15
G-IV (Standard: Glibenclamide)	10 mg/kg b.w. (oral)	4.04 ± 0.10	2.02 ± 0.15^†^	49.25

Mean ± S.E.M. (*n*=5). Data were analyzed using one-way ANOVA followed by Dunnett’s *t* test and compared with control.

* *P*<0.01.

† *P*<0.005 was considered significant.

#### Assessment of OGTT of ZnONPs

**Figure 3:**
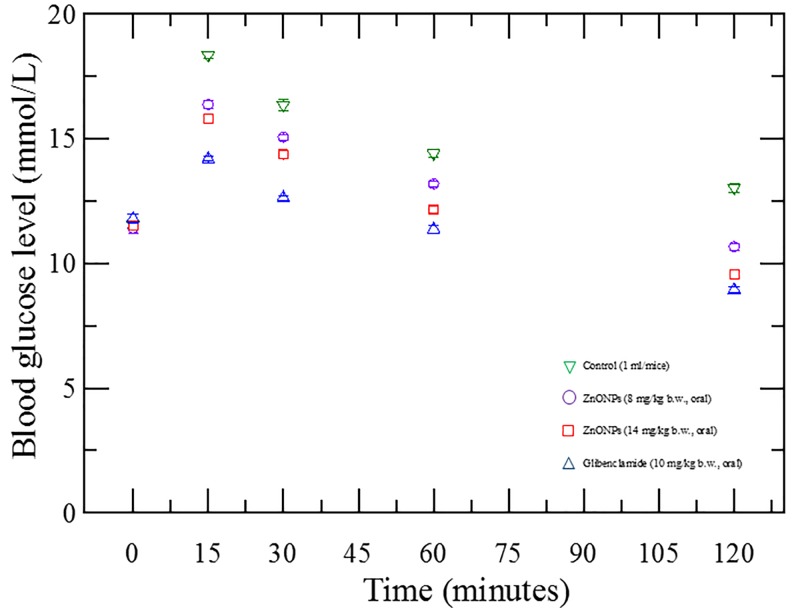
Oral glucose tolerance effect of ZnONPs The figure shows that ZnONPs have a significant blood glucose levels lowering activity and the response follows dose-dependent manner(s).

ZnONPs (8 mg/kg b.w. for G-II and 14 mg/kg b.w. for G-III mice) significantly reduced the blood glucose levels at 15, 30, 60 and 120 min time interval when compared with control (G-I). Glibenclamide (G-IV) was used as a standard in this experiment. The treatment of ZnONPs resulted in suppression of blood glucose levels of mice is shown in Figure 3.

## Discussion

Nanoparticles of many metals such as: zinc, silver, iron and gold oxides have revealed a significant role in medical and biological applications [26]. In this experiment, the administration of ZnONPs to diabetic mice resulted in a considerable reduction in fasted blood glucose levels. The key mechanism of hyperglycemia involves over-production (hepatic glycogenolysis and gluconeogenesis) and decreased utilization of glucose by the tissues [27]. Our result revealed that ZnONPs significantly reduced the glucose levels in STZ-induced diabetic mice. Almost 39.74 and 38.78% reduction in blood glucose levels were found for the groups of mice- ZnONPs, (ZnONPs + insulin), respectively (Table 1) when compared with the control group. STZ may enter the β-cell via a glucose transporter and causes alkylation of DNA [28]. DNA damage induces activation of poly-ADP-ribosylation, a process that is more important for the diabetogenicity of STZ than DNA damage itself. Zinc may provide insulin-like effects in the signal transduction mechanism of insulin and reduce the production of cytokines, which leads to β-cell death due to inflammation in the pancreas during diabetes [13]. According to the study of Alkaladi et al. (2014) [21], ZnONPs can increase serum insulin levels and increase the expression of mRNA of the insulin gene. Other studies found that glucose-stimulated insulin secretion is enhanced by zinc through isolated pancreatic islets of rats [29]. Here ZnONPs may play a role in improving the glucose utilization and metabolism through influencing the improvement of hepatic glycogenesis. ZnONPs may act on the insulin signaling pathway in this aspect [30]. Our study is found to be consistent with the finding of Alkaladi et al. (2014) [21].

The hypoglycemic activity test was done by using two doses of ZnONPs (8 mg/kg and 14 mg/kg b.w. of mice). ZnONPs showed a positive result in hypoglycemia test. It reduced the blood glucose levels significantly (25.13%) for mild dose and (29.15%) for a moderate dose. The standard drug glibenclamide reduced (49.25%) blood glucose levels. It is well established that sulphonylureas (e.g., glibenclamide) cause hypoglycemia by stimulating insulin release from pancreatic β-cell [31]. The comparable effect of the ZnONPs with glibenclamide suggests the possibility of a similar mode of action.

In OGTT, the serum glucose levels of normal mice reached a peak significantly after 15 min nONPs dosing. Mice were given glucose orally (2 g/kg b.w. of mice) just a few minutes before the ZnONPs sampling. We found that blood glucose levels decrease gradually within 2 h. A single administration of ZnONPs dose resulted in prominent glucose suppression during OGTT, which confirm the possibility of antidiabetic effect. The results of our study endorse the finding of Umrani and Paknikar (2014) [22].

Many *in vitro* and *in vivo* experiments have verified that zinc has beneficial effects in both type-1 and type-2 diabetes. It is discernible from the findings of the study that zinc plays an essential role in β-cell functioning, glucose homeostasis, the pathogenesis of diabetes and diabetes complications. The purpose of zinc in diabetes has been established by supplementation studies in diabetic rats [32]. Several zinc complexes were synthesized, and they are proven to be very useful in the biological system [33,34]. Many types of research demonstrate that zinc is secreted along with insulin served as an autocrine molecule which enhances the glucose-stimulated insulin secretion from the rat-isolated pancreatic islets [29]. Our experiments revealed that ZnONPs lowered blood glucose levels in diabetic mice significantly. Based on our experimental results, we can suggest that ZnONPs might act as an insulin secretagogue. In the single-dose OGTT study, experimental mice showed an elevated blood glucose levels which reduced after 2 h. Similarly, ZnONPs dose reduced the blood glucose levels just like the antidiabetic action of ZnONPs. As STZ mimic the effect of type-1 diabetes, there is less amount of β-cell mass in the pancreas.

Further, zinc has been reported to regulate gluca­gon secretion from pancreatic α-cells [35]. Consequently, glucagon-stimulated hepatic pathways (i.e., gluconeogenesis, glycogenesis) would be inhibited in the fasting state of mice which may lead to reduced blood glucose levels after zinc dosing. In the case of hypoglycemic action, the fasting blood glucose levels in healthy mice were in a control state; however, the single-dose administration of ZnONPs significantly reduced the blood glucose levels. Glibenclamide is used in both hypoglycemic and glucose tolerance test as a controlled drug, and it is used in type-2 diabetes. The test groups (G-II, G-III) significantly reduced blood glucose level; that means ZnONP has a significant role in controlling Type-2 diabetes by reducing the production of inflammatory cytokines [13]. Comparing this point of view, ZnONPs may be a potential candidate of agent for controlling Type-2 diabetes. This may be due to the activation of another physiological pathway such as insulin secretion, glucagon regulation and accumulation of glucose in the liver and adipose tissue. Zinc transporters are also recognized in adipose tissues and liver as these organs are the critical regulator of glucose metabolism [36].

## Conclusion

Considering the data, ZnONPs showed promising antidiabetic activity, which could be potential in developing an antidiabetic drug. However, this study did not investigate the physiological parameters and molecular mechanisms of ZnONPs; therefore, further mechanistic studies are warranted to supports its antidiabetic action in mice.

## Abbreviations

DM: diabetes mellitus
OGTT: oral glucose tolerance test
SEM: scanning electron microscopy
STZ: streptozotocin
TEM: transmission electron microscopy
ZnONP: zinc oxide nanoparticle
